# Akt/FoxM1 signaling pathway-mediated upregulation of MYBL2 promotes progression of human glioma

**DOI:** 10.1186/s13046-017-0573-6

**Published:** 2017-08-07

**Authors:** Xue Zhang, Qiao-Li LV, Yuan-Tao Huang, Li-Hua Zhang, Hong-Hao Zhou

**Affiliations:** 1 Department of Clinical Pharmacology, Xiangya Hospital, Central South University, Changsha, 410008 Hunan People’s Republic of China; 2 Institute of Clinical Pharmacology, Central South University; Hunan Key Laboratory of Pharmacogenetics, Changsha, 410078 Hunan People’s Republic of China; 3 Department of Neurology, The Brain Hospital of Hunan Province, Changsha, 410007 Hunan People’s Republic of China

**Keywords:** Glioma, FoxM1, MYBL2, Progression

## Abstract

**Background:**

MYB-related protein B (B-MYB/MYBL2), a member of the myeloblastosis family of transcription factors, has been reported for its role in the genesis and progression of tumors. Forkhead box M1 (FoxM1), another transcriptional factor, is considered to be an independent predictor of poor survival in many solid cancers. The aim of the present study was to investigate the clinical significance of the correlation between MYBL2 and FoxM1 in glioma and the possible mechanism of FoxM1and MYBL2 expression.

**Methods:**

MYBL2 and FoxM1expression in cancerous tissues and cell lines were determined by reverse transcription-PCR (RT-PCR), Western blotting and immunostaining. The co-expression of MYBL2 and FoxM1 was analyzed in low-grade glioma (LGG) and glioblastoma (HGG) cohorts of TCGA using cBioportal and UCSC Xena. And, the role of MYBL2 and FoxM1 in glioma cell progression and the underlying mechanisms were studied by using small interfering RNA (si-RNA) and pcDNA3.1 + HAvectors. Furthermore, the effects of MYBL2 and FoxM1 in cell proliferation, cell cycle progression, apoptosis, migration, invasion, and adhesion were determined by cell proliferation assays, flow cytometry analysis, transwell migration and cell adhesion assay.

**Results:**

MYBL2 and FoxM1 expression are significantly associated with clinical stages and overall survival of glioma patients. In cohorts of TCGA, patients with high MYBL2 but without radio-chemotherapy had the highest hazard ratio (adjusted HR = 5.29, 95% CI = 1.475–18.969, *P* < 0.05). Meanwhile, MYBL2 closely related to the FoxM1 expression in 79 glioma tissues (*r* = 0.742, *p* < 0.05) and LGG (*r* = 0.83) and HGG (*r* = 0.74) cohorts of TCGA. Down regulation of FoxM1 and MYBL2 by siRNAs induced the cell cycle arrest, apoptosis and EMT of glioma cells. Furthermore, inactivations of Akt/FoxM1 signaling by Akt inhibitor and siRNA-FoxM1 reduce the expression of MYBL2 in glioma cells.

**Conclusions:**

MYBL2 is a key downstream factor of Akt/FoxM1 signaling to promote progression of human glioma, and could be a new candidate gene for molecular targeting therapy and biomarker for radiotherapy of glioma.

**Trial registration:**

*CTXY-1300041-3-2*. ChiCTR-COC-15006186. Registered date: 13 September 2013.

**Electronic supplementary material:**

The online version of this article (doi:10.1186/s13046-017-0573-6) contains supplementary material, which is available to authorized users.

## Background

Gliomas are the most common form of primary brain tumor in the adult central nervous system [1]. Although recent studies showed progress in both diagnostic modalities and therapeutic strategies, glioma remains one of the deadliest human cancers. The five-year survival rate of patients with glioma is the lowest among those of all cancers [2, 3]. The alternative treatment for glioma is limited due to the unclear pathophysiological mechanisms underlying the development of this disease. Therefore, understanding the molecular mechanism of the development and progression of glioma will shed light on strategies for accurate diagnosis, early intervention, and effective therapies.

Transcription factors (TFs) play important roles in the transcriptional networks that regulate gene expression, and misregulation of these TFs can result in the acquisition of tumor-related properties [4]. Differentially expressed TFs in glioblastoma, and their downstream gene targets may be potential therapeutic biomarkers of glioblastoma [5]. The MYBL2 gene, which is also known as B-MYB, is a member of the myeloblastosis family of transcription factors, first identified as cellular homologues of the *v-myb* oncogene that is known to cause leukemia in chickens [6]. MYBL2 of proliferative cells is crucial for the regulation of proliferation and differentiation, and also has a vital role in guiding cell cycle progression [7]. Meanwhile, MYBL2 amplification or overexpression has been observed in cancers such as myeloid leukemias (AML) [8], hepatocellular carcinoma [9], breast cancer [10], and it is currently used as a marker for poor prognosis in colorectal carcinoma [11]. However, it is still not clear about the role of the MYBL2 gene in glioma.

FoxM1 is a member of the Forkhead box (Fox) transcription factor family, which has been shown to be over-expressed in various cancers and studies have shown that alterations in FoxM1 signaling were associated with carcinogenesis. FoxM1 is substantially elevated in several aggressive human carcinomas and can contribute to oncogenesis in many tissue types, including breast [12], hepatocellular [13], prostate [14], lung [15], and colorectal cancers [16]. Aberrant FoxM1expression was found to be a common molecular alteration in malignant glioma [17]. Moreover, it has been shown that higher expression of FoxM1 was associated with poor prognosis and radio resistance in glioma patient [18–20]. Previous studies showed that FoxM1 was a key downstream gene of the Akt/FoxM1 signaling cascade [21]. Another finding suggested that Akt/FoxM1 signaling played an impotent role in cervical cancer cell growth and treatment [22]. However, the role and mechanism of Akt/FoxM1 signaling in the development of glioma is unknown.

Here, we have thoroughly investigated the expression levels of MYBL2 in glioma tissues and cell lines. And, we sought to determine whether Akt/FoxM1 signaling pathway is involved in regulating MYBL2 expression and whether these factors can predict the disease progression and prognosis of the glioma patients.

## Methods

### Patient samples

All samples, along with available clinical-pathological data, were obtained from were obtained from Xiangya Hospital of Central South University (Changsha, Hunan, China) between 2013 and 2014, with written informed consent. All pathological features were confirmed by experienced pathologists, and none of the patients received pre-operative anti-cancer treatment. All procedures were approved by the Ethics Committee Institute of Clinical Pharmacology, Central South University (Ethical Approval No. *CTXY-1300041-3*). These patients 79 had completed a follow-up time along with 48 months from the date of surgical resection. Overall survival (OS) was defined as a period time between the date of the initial surgical operation and death or the last follow-up.

#### Bioinformatic data mining

We downloaded RNA-Seq gene expression data (Level 3) and clinical data from the TCGA data portal (https://gdc-portal.nci.nih.gov/) as our other source of samples, and a total of 567 tumors having clinical data were profiled for class discovery and survival analysis [23]. For overall survival (OS) and relapse-free survival (RFS) were searched in the glioma patient cohort in TCGA database using cBioportal (http://cbioportal.org). The heat map and the correlation betweenMYBL2 and FoxM1 genes in the same patient cohort were further verified and analyzed using UCSC Xena t (http://xena.ucsc.edu/). Moreover, the molecular functional network map of canonical pathways including coexpression, physical interaction, and predicted networks of FoxM1 analyzed by GeneMANIA (http://genemania.org/).

### Cell culture

Penicillin, streptomycin, trypsin-EDTA (ethylene diaminetetra acetic acid) were purchased from Beyotime (Beijing, China). The human glioma cell lines U251, U343, U87, T98G, and Hs683 were obtained from the Type Culture Collection of the Chinese Academy of Sciences (Shanghai, China). Cells were cultured in and cultured in DMEM (Invitrogen, Shanghai, China), containing 10% fetal bovine serum (Gibco, Logan, UT, USA) and 100 U penicillin and streptomycin at 37 °C in a humidified atmosphere containing 5% CO_2_.

### Cell transfection

Three different interfering RNA (siRNA) for the specific inhibition of MYBL2 and FoxM1 expression and a negative control siRNA were synthesized by GenePharma Co., Ltd. (Shanghai, China). Exponentially growing untreated cells were plated 24 h before transfection. Plated cells were transfected with FoxM1 siRNA or MYBL2 siRNA at a final concentration of 50 nM, using 5 μL Lipo-RNAiMAX following the manufacturer’s instruction (Invitrogen, USA). After treatment, the cells were harvested and processed for further analysis. Human FoxM1 gene was inserted in pcDNA3.1 + HAvector and MYBL2 was inserted in GV230- GFP by Life Technologies (Shanghai Genechem, Co., Ltd., Shanghai, China) and the empty vector was used as the negative control. Hs683 cells transfected with FoxM1 and MYBL2 vectors with Lipofectamine 2000 according to the manufacturer’s information.

### RNA extraction and quantitative real-time PCR (RT-qPCR)

Total RNA was extracted from cultured cells or tissue samples using TRIzol reagent (Invitrogen, Carlsbad, CA, USA) according to the manufacturer’s instructions. First-strand cDNA synthesis was performed using Prime Script RT Master Mix (TaKaRa Biotechnology Co., Ltd., Dalian, China). Real-time quantitative PCR was performed using a standard SYBR Green PCR kit (Thermo). All reactions were conducted using the following cycling parameters: 95 °C for 2 min, followed by 40 cycles of 95 °C for 15 s, with a final extension at 60 °C for 60 s. GAPDH was used as an endogenous control. The gene expression was calculated using the ΔΔCt method. All data represent the average of three replicates. The primers used are listed in Table 1.

**Table 1:** Oligonucleotide primer sequences used in the qRT-PCR

Gene	Forward	Reverse
MYBL2	5’-CTTGAGCGAGTCCAAAGACTG-3’	5’-AGTTGGTCAGAAGACTTCCCT-3’
FOXM1	5’-ATACGTGGATTGAGGACCACT-3’	5’-TCCAATGTCAAGTAGCGGTTG-3’
Akt	5’-GACTACCTGCACTCGGAGAAG-3’	5’-TGTGATCTTAATGTGCCCGTC-3’
GAPDH	5’-CCCATCACCATCTTCCAGGAG-3’	5’-CTTCTCCATGGTGGTGAAGACG-3’
β-actin	5’-CATGTACGTTGCTATCCAGGC-3’	5’-CTCCTTAATGTCACGCACGAT-3’

The specific oligonucleotide primer sequencesare listed in Table 1. GAPDH and β-actin were used as an internal controls and the qRT-PCR result was quantified by 2–ΔΔCT method

### Cell viability and proliferation assays

Cell viability and proliferation were measured by MTT assay after treatment. The identified cells were seeded in 96-well plates (6 × 10^3^ cells/ well) and transfected with siRNAs. After culturing cell for an appropriate time, 50 μL of 5 mg/ml MTT (Sigma) was added to each well and cultured for 4 h. Then, the cell culture medium was replaced by 100 μL of dimethyl sulfoxide. After 2–3 h of incubation at 37 °C, the number of viable growing cells was estimated by measuring absorption at 570 nm wavelength and cell growth curves were determined according to the optical density value. The proliferation rate was calculated using the following formula: proliferation rate = survival rate = (OD test/ OD negative control) × 100%. All experiments were performed in triplicate and repeated at least three times.

### Cell cycle analysis

Propidium iodide (PI) staining was used to analyze DNA content. Treated and untreated cells were harvested and labeled with PI by using previously described methods. Briefly, Cells in each group were washed with PBS for twice and centrifuged at 5000 rpm for 7 min to regulate the density as 1 × 10^6^ cells/ well. Then, pre-cooling 70% ethyl alcohol was added for fixation overnight at −20 °C. On the next day, the fixed cells were washed with PBS, incubated with 400 μl PI/ RNase Staining Buffer (BD Company) at room temperature in the dark for 15 min. The cell cycle distribution was determined using a flow cytometer (Beckman Coulter, Brea, CA, USA). We then determined the percentage of cells in the G0/G1, S, and G2/M phases with the FlowJo software (Tree Star). The experiment was repeated for three times.

### Apoptosis analysis

Apoptosis was assessed by Annexin V staining and flow cytometry analysis. Briefly, 3 × 10^5^ cells were harvested, washed in PBS, and then analyzed by Annexin V/ propidium iodide staining according to the manufacturer’s protocol (FITC-Annexin V kit; BD Pharmingen, San Diego, CA). The stained cells were analyzed by flow cytometry.

### Colony formation and clonogenic assays

U251 cells were seeded in 6-well plates (1.5 × 10^3^ cells/ well); transfected with a non- silencing control siRNA, MYBL2 siRNA, or FoxM1 siRNA. After 15 days of incubation in the incubator, cell colonial forming amount was observed under the inverted microscope. And then, the cells were washed with PBS and stained with crystal violet, and visible colonies were counted.

### Cell migration and motility

Cells were seeded in six-well plates (5 × 10^5^ cells/well) and 24 h later were transfected with the control siRNA, MYBL2 siRNA (50 nM) or FoxM1 siRNA (50 nM). After culturing cell for an appropriate time, artificial wounds were gently made using a micropipette tip, and the cells were washed with PBS to remove floating cells and debris. The cells were then incubated in serum-free medium. Cells in the scratched area were imaged at 0 and 48 h using microscopy, and the distance traveled by cells at the leading edge of the wound at each time point was measured. The results were expressed as percent migration.

### Transwell migration and invasion assays

Cell migration and invasion were assessed using a transwell assay. For migration assays, Matrigel (1:8) (BD Biosciences, Bedford, MA, USA) was diluted with serum-free DMEM, and the basement membrane of the upper chamber of the transwell was coated. The solution was kept at 37 °C for 1–4 h to transform the Matrigel aggregate into the gel. Treated cells were harvested and dilution with serum-free DMEM (5 × 10^5^ cells/mL) 200 μL was added to a transwell insert (pore size, 8 μm; BD Biosciences, San Jose, CA, USA), and 600 μL containing 20% FBS was added to the lower chamber. Cells at each concentration were cultured in a 24-well plate in a 5% CO_2_ incubator at 37 °C for 24 h [24]. The culture medium in each well was then discarded, and the chamber was washed twice with PBS. And gently removing the cells in the upper chamber with a cotton swab, the cells on the underside of the membrane were fixed with 4% paraformaldehyde for 15 min, stained with 0.1% cresyl violet, washed three times with PBS, and air-dried. Five fields (200 × magnification) were randomly selected for counting the number of migrated cells, and images were taken by using phase contrast microscopy.

### Cell adhesion assay

Ninety-six-well dishes were pre-coated with 30 mg/L fibronectin solution (50 μL/well), then air-dried at room temperature overnight, and then rinsed with PBS and incubated with 3% heat-denatured BSA to block any uncoated areas. Cells were cultured in a 5% CO_2_ incubator at 37 °C for 1 h. The culture solution was then removed from the 24-well plate, and non-adherent cells were washed away three times with PBS. The remaining cells were fixed for 30 min with 4% paraformaldehyde, stained with cresyl violet for 15 min, and observed under an inverted microscope.

### IHC

TMA slides were processed and stained manually as described previously. Formalin- fixed, paraffin-embedded sections were prepared for all tissues. Sections were deparaffinized in xylene and rehydrated through graded alcohol to water, and endogenous peroxidase activity was blocked by incubating the slides in 3% H_2_O_2_ in water for 30 min at room temperature. Sections were incubated in 1% BSA for 30 min then wiped off and dilution of MYBL2 (1:200, Santa Cruz) and FoxM1 (1:200, Santa Cruz) were applied to the slides and incubated overnight at room temperature. Subsequently, sections were incubated with secondary antibody for 2 h at room temperature, according to the manufacturer’s instructions. Negative control slides were processed in parallel using a non-specific immunoglobulin IgG (Sigma Chemical Co, St. Louis, MO, USA) at the same concentration as the primary antibody. Stained sections were observed under a microscope. Only fresh cut slides were stained simultaneously to minimize the influence of slide ageing and maximize repeatability and reproducibility of the experiment.

### Immunofluorescence double staining

U251 cells were planted on glass slides in a 6-well at a density of 1 × 10^6^ cells per well. Treated and untreated cells were fixed with ice-cold 4% paraformaldehyde for 30 min, permeabilised with 0.1% Triton X-100, and blocked in 2% gelatin in PBS at room temperature. Cells were then incubated with MYBL2 (Millipore, USA, 1:500) and FoxM1 (Cell Signaling Technology, USA, 1: 500) primary antibodies at 4 °C overnight. After being washed, the cells were incubated with secondary antibody (1:100) for 1 h at room temperature. The cell nucleus showed blue fluorescence (stained by DAPI). Images were obtained under a fluorescence microscope.

### Western blot analysis

Cells from each group were collected, and whole-cell lysates were generated using RIPA lysis buffer (Abcam, Cambridge, UK). The protein concentration was detected by BCA method (U.S. Pierce Company). Total proteins were separated using 10% or 12% SDS-PAGE and then transferred onto a nitrocellulose membrane. The membrane was incubated with the primary antibody at 4 °C overnight, followed by a horseradish peroxidase-conjugated secondary antibody the next day for 2 h at room temperature. β-actin and GAPDH were purchased from (Sigma, USA). MYBL2 was purchased from (Millipore, USA). P-Akt and p-38 MAPK were purchased from (Santa Cruz Biotechnology, USA). SC79 (Akt activator) and MK-2206 (Akt inhibitor) were purchased from Selleck (Beijing, China). E-Cadherin(Wanleibio, China). Cyclins (CDK2, CDK6, CyclinD1, CyclinD3, CDK4, etc.), FoxM1, ZEB1, MMP9, MMP2, Vimentin and N- Cadherin were purchased from Cell Signaling Technology (Danvers, MA, USA).

### Statistical analysis

Statistical significance between different groups was determined using t-tests for the MYBL2 and FoxM1 mRNA levels analysis and cell assay. Kaplan-Meier survival curves were used to compare survival rates. Univariate and multivariate Cox proportional hazard models were used to explore the associations between patient characteristics and biomarkers with outcomes. Statistical analyses were performed using SPSS19.0 software (IBM, Chicago, IL, USA). Data are shown as mean ± standard deviation. *P*-values <0.05 differences was considered statistically significant.

## Results

### Association of MYBL2 and FoxM1 with glioma risk

**Fig. 1:**
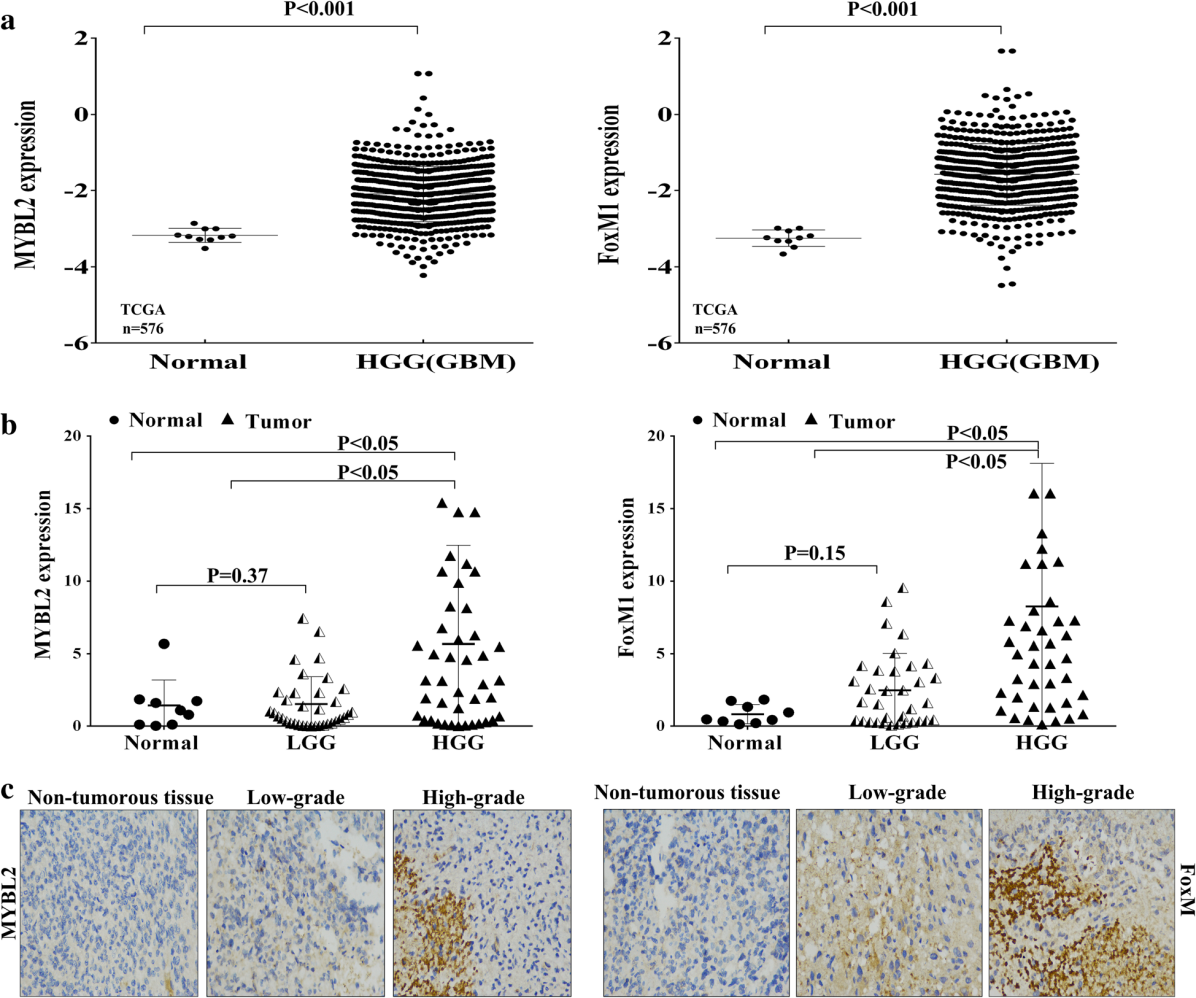
Relative expression of MYBL2 and FoxM1 in glioma tissues. **a** MYBL2 and FoxM1 expression in TCGA dataset. **b** MYBL2 and FoxM1 mRNA expression by RT-qPCR in 79 primary glioma tissues and 9 normal brain tissues. **c** FoxM1 and MYBL2 protein expression levels in 1 normal and 26 glioma tissues by using IHC

To determine whether MYBL2 and FoxM1 were differentially expressed between glioma and normal tissues, the mRNA expressions were analyzed by qRT-PCR in 79 glioma and 9 normal tissues. Both MYBL2 and FoxM1 levels were significantly higher in glioma than in normal tissues, similar to the IHC results (Fig. 1b and c). We then divided the glioma patients into different groups based on the mean value (3.83) of relative MYBL2 and the mean value (5.88) of relative FoxM1 expression. We found that MYBL2 and FoxM1 expression had a close correlation with the tumor progression: both genes were higher expression in patients with advanced tumor stage than in normal tissues (*P < 0.05) and early-stage (*P < 0.05). But the tumor progression was not correlated with patient’s age, gender and tumor location (*P* > 0.05, Tables 2 and 3).

**Table 2:** Correlation between MYBL2 expression and clinicopathological features in 79 glioma patients

Clinical characteristic	NO. of patients	NO. of patients		*P* Valaue
		High expression(*n*=24)	Low expression(*n*=55)	
Age (year)				
≧45	30	9	21	0.474524
<45	49	15	34	
Sex				
Male	50	15	35	0.329518
female	29	9	20	
Clinical Stage				
Low grades I-II	35	4	31	<0.001
High gradesIII - IV	44	20	24	
Tumor location				
Frontal	34	10	24	0.738
Parietal	13	5	8	
Occipital	1	1	0	
Temporal	18	4	14	
Others	13	4	9

**Table 3:** Correlation between FoxM1 expression and clinicopathological features of glioma patients

Clinical characteristic	NO. of patients	NO. of patients		*P* Valaue
		High expression(*n*=26)	Low expression(*n*=53)	
Age (year)				
≧45	30	13	17	0.102647
<45	49	13	36	
Sex				
Male	50	16	34	0.210681
female	29	10	19	
Clinical Stage				
Low grades I-II	35	5	30	<0.01
High gradesIII - IV	44	21	23	
Tumor location				
Frontal	34	10	24	0.357
Parietal	13	4	7	
Occipital	1	0	1	
Temporal	18	6	12	
Others	13	6	7	

To further confirm the association of MYBL2 and FoxM1 with glioma risk**,** we analyzed the gene expression data of glioma cases in the high-grade glioma (HGG) TCGA data set, which includes 567 glioma tissues and 10 non-tumor tissues. MYBL2 and FoxM1 mRNA expression were found to be significantly increased (MYBL2, *P* < 0.001; FoxM1, *P* < 0.001) in HGG compared to normal brain (Fig. 1a). The characteristics and clinical features of 567 high-grade glioma patients are shown in Additional file 1: Table S1.

### MYBL2 and FoxM1 overexpression linked with poor outcome

**Fig. 2:**
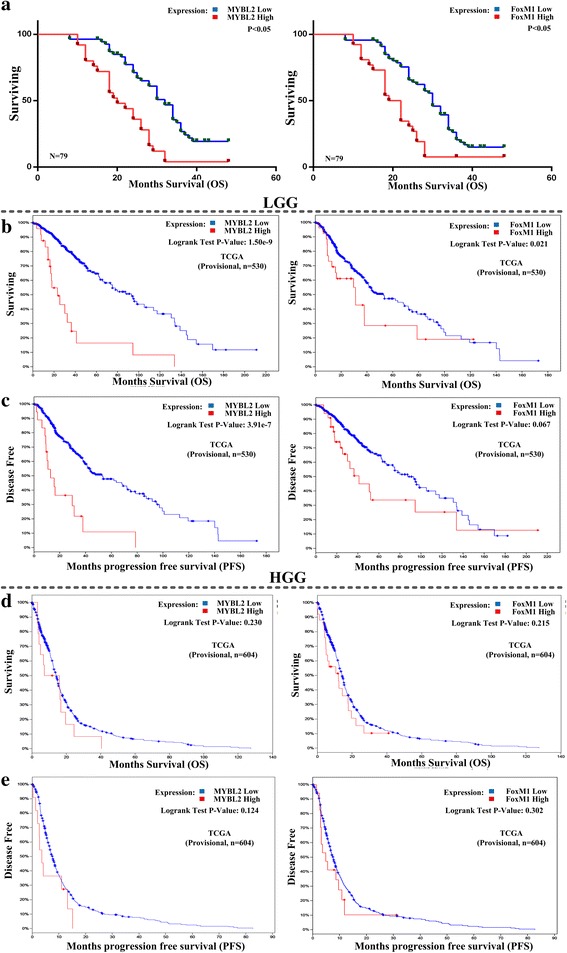
Survival analyses of cancer patients based on expression of MYBL2 and FoxM1. **a** Compare overall survival time between MYBL2 (left) or FoxM1 (right) higher expression levels and lower-expression-level in 79 glioma tissues. **b** Compare overall survival time between MYBL2 (left) or FoxM1 (right) higher expression levels and lower expression levels in LGG. **c** Associations between MYBL2 (left) and FoxM1 (right) gene expression levels and disease-free survival in LGG. **d** Compare overall survival time between MYBL2 (left) or FoxM1 (right) higher expression levels and lower-expression-level in HGG. **e** Associations between MYBL2 (left) and FoxM1 (right) gene expression levels and disease-free survival in HGG

We evaluated the effects of MYBL2 and FoxM1 on overall survival of the glioma patients using Kaplan-Meier analysis and log-rank test. In 79 glioma cases, MYBL2 and FoxM1 expression were significantly associated with glioma patients’ overall survival (OS) (MYBL2, *P* < 0.001; FoxM1, *P* < 0.001, Fig. 2b). Univariate Cox regression analysis indicated that the clinical stage (HR = 1.833, 95% CI: 1.395–2.409, *p* < 0.001) and high expression of MYBL2 (HR = 3.619, 95% CI: 2.075–6.313, *p* < 0.001) and FoxM1 (HR = 0.336, 95% CI: 0.187–0.602, *p* < 0.001) were unfavorable prognostic factor in glioma patients (Tables 4 and 5).

**Table 4:** Univariate and multivariate Cox regression of MYBL2 for overall survival in glioma

	OS			
Variable	Univariate analysis		Multivariate analysis	
	HR (95% CI)	*P*-value	HR (95% CI)	*P*-value
Age (year)				
<45 vs. ≥45	0.874 (0.488-1.567)	0.652	0.873 (0.474-1.606)	0.873
Gender				
Female vs. male	1.038 (0.617-1.746)	0.889	1.021 (0.590-1.767)	0.941
Clinical Stage				
III-IV vs. I-II	1.833(1.395-2.409)	<0.001	0.360 (0.193 -0.670)	<0.05
Tumor Location				
Parietal vs. Frontal	1.413 (0.715-2.790)	0.32	0.731 (0.324 -1.650)	0.45
Temporal vs. Frontal	1.173 (0.652-2.110)	0.594	0.995 (0.517-1.914)	0.966
Others vs. Frontal	0.605 (0.297-1.232)	0.166	0.335 (0.308-1.493)	0.301
MYBL2 Expression				
Low vs. high	3.619 (2.075-6.313)	<0.001	0.354 (0.193-0.650)	<0.05

*HR* hazard ratio; *CI* confidence interval; *MYBL2* MYB-related protein B

**Table 5:** Univariate and multivariate Cox regression of FoxM1 for overall survival in glioma

	OS			
Variable	Univariate analysis		Multivariate analysis	
	HR (95% CI)	*P*-value	HR (95% CI)	*P*-value
Age (year)				
<45 vs. ≥45	0.874 (0.488-1.567)	0.652	0.964 (0.528-1.761)	0.905
Gender				
Female vs. male	.964 (0.573 -1.622)	0.889	1.010 (0.585-1.744)	0.971
Clinical Stage				
III-IV vs. I-II	0.297 (0.172-0.514)	<0.001	0.347 (0.188-0.642)	<0.05
Tumor Location				
Parietal vs. Frontal	0.708 (0.358-1.398)	0.32	1.068 (0.465-2.454)	0.876
Temporal vs. Frontal	0.852 (0.474-1.533)	0.594	1.170 (0.589-2.321)	0.654
Others vs. Frontal	1.653 (0.812-3.364)	0.166	1.425 (0.651-3.116)	0.3754375
FoxM1 Expression				
Low vs. high	0.336 (0.187-0.602)	<0.001	0.391 (0.196-0.779)	<0.05

*HR* hazard ratio, *CI* confidence interval, *FoxM1* Forkhead box M1

To confirm the association of these gene signatures with the outcome, we compared OS (overall survival) and DFS (disease free survival) between patients with higher expression levels and patients with lower expression levels of MYBL2 and FoxM1 genes in low-grade glioma (LGG) and glioblastoma (HGG) cohorts of TCGA using cBioPortal. Kaplan-Meier survival curves show that patients with lower expression levels of MYBLL2 or FoxM1 have better OS and DFS prognoses than those with higher expression levels in LGG group (Fig. 2b and c, log-rank test, unadjusted *P*-value <0.05). Though there is no significant difference, patients with lower expression levels of MYBL2 or FoxM1 have better OS and DFS prognoses than those with higher expression levels (Fig. 2d and e, log-rank test, unadjusted *P*-value >0.05). These results indicated that low expression of MYBL2 and FoxM1 probably confer a survival advantage to glioma patients.

### MYBL2 is a radiosensibility biomarker of glioma

To further characterize the association of MYBL2 and FoxM1with glioma survival, we analyzed the interaction of MYBL2 and FoxM1 with radiotherapy status in HGG cohorts of TCGA, and observed that compared to patients with MYBL2 over-expression and radiotherapy, those with MYBL2 over-expression but without radiotherapy had a significantly higher death risk (adjusted HR = 5.29, 95% CI = 1.475–18.969, *P* < 0.05) (Tables 6 and 7). These results suggesting that in high-grade glioma, MYBL2 gene over-expression might identify patients who will not benefit from the treatment of radiotherapy.

**Table 6:** Interaction between MYBL2 expression and radiotherapy on HGG glioma survival

MYBL2 expression	Radiotherapy	Patients	Deaths	MST(Months)	Adjusted HR (95% CI)	P*
High	Yes	136	127	4.9	1	
High	No	404	292	9.6	5.29 (1.475-18.969)	0.011*
Low	Yes	10	8	1.5	0.995 (0.335-2.958)	0.993
Low	No	17	12	7.7	1.769 (0.267-11.697)	0.554

*p-vale<0.05; Abbreviations: *MST* median survival time

Adjusted for age, gender, race, and history neoadjuvant treatment

**Table 7:** Interaction between FoxM1 expression and radiotherapy on HGG glioma survival

FoxM1 expression	Radiotherapy	Patients	Deaths	MST(Months)	Adjusted HR (95% CI)	P*
High	Yes	143	133	4.7	1	
High	No	413	298	9.7	5.486 (0.939-32.043)	0.05
Low	Yes	3	2	1.3	0.998 (1.91-5.217)	0.99
Low	No	8	6	3.8	0.76(0.41-14.072)	0.85

*p-vale<0.05; Abbreviations: *MST* median survival time

Adjusted for age, gender, race, and history neoadjuvant treatment

### Altering the expression of MYBL2 and FoxM1 in glioma cells

**Fig. 3:**
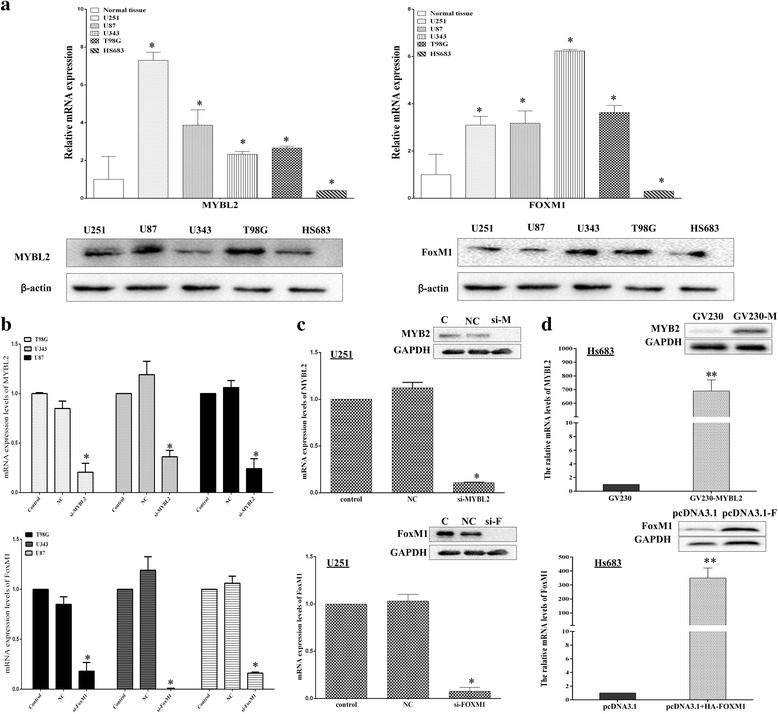
Altering expression of FoxM1 and MYBL2 mRNAs and proteins in glioma cell lines. **a** The expression of mRNAs and proteins of MYBL2 and FoxM1 in Glioma cell lines. **b** T98G, U343 and U87 cells were transfected with MYBL2-specific siRNA (upper) and FoxM1-specific siRNA (lower) for 24 h. **c** U251cells were transfected with MYBL2-siRNA (upper) and FoxM1-siRNA (lower). **d** Hs683 cells were transfected with GV230-MYBL2 (upper) pcDNA3.1 + HA-FOXM1 (lower). The relative mRNA and protein expression levels were measured. *P values <0.05; ** p values <0.001﻿

We performed qRT-PCR analysis and Western blotting to test FoxM1 and MYBL2 expression in high-grade glioma cell lines (U251, U87, U343 and T98G), low-grade cell line (Hs683) and 9 normal tissues. As shown in Fig. 3a (upper), all of the high-grade cell lines exhibited higher mRNA expression of FoxM1 and MYBL2 compared to the normal tissues. Similar results were found in protein level (Fig. 3a).

To investigate the functions of MYBL2 and FoxM1 expression in glioma, we up and down regulated both genes in low and high-grade glioma cells. Firstly, we transected GV230-MYBL2 (2 μg/mL) and pcDNA3.1 + HA-FOXM1 (2 μg/mL) to increase the genes expression in low grade glioma Hs683 cells, respectively. The transfection efficiency of the plasmid vectors was evaluated by real-time PCR and Western blotting (Fig. 3d). Then, we knocked down both genes expression by RNA interference (RNAi) in high-grade glioma cells, including U87, T98G, U343 and U251 cells. The silencing effects of the siRNA were also evaluated by real-time PCR and Western blotting (Fig. 3b and c).

### MYBL2 and FoxM1 accelerate tumor progression in glioma

**Fig. 4:**
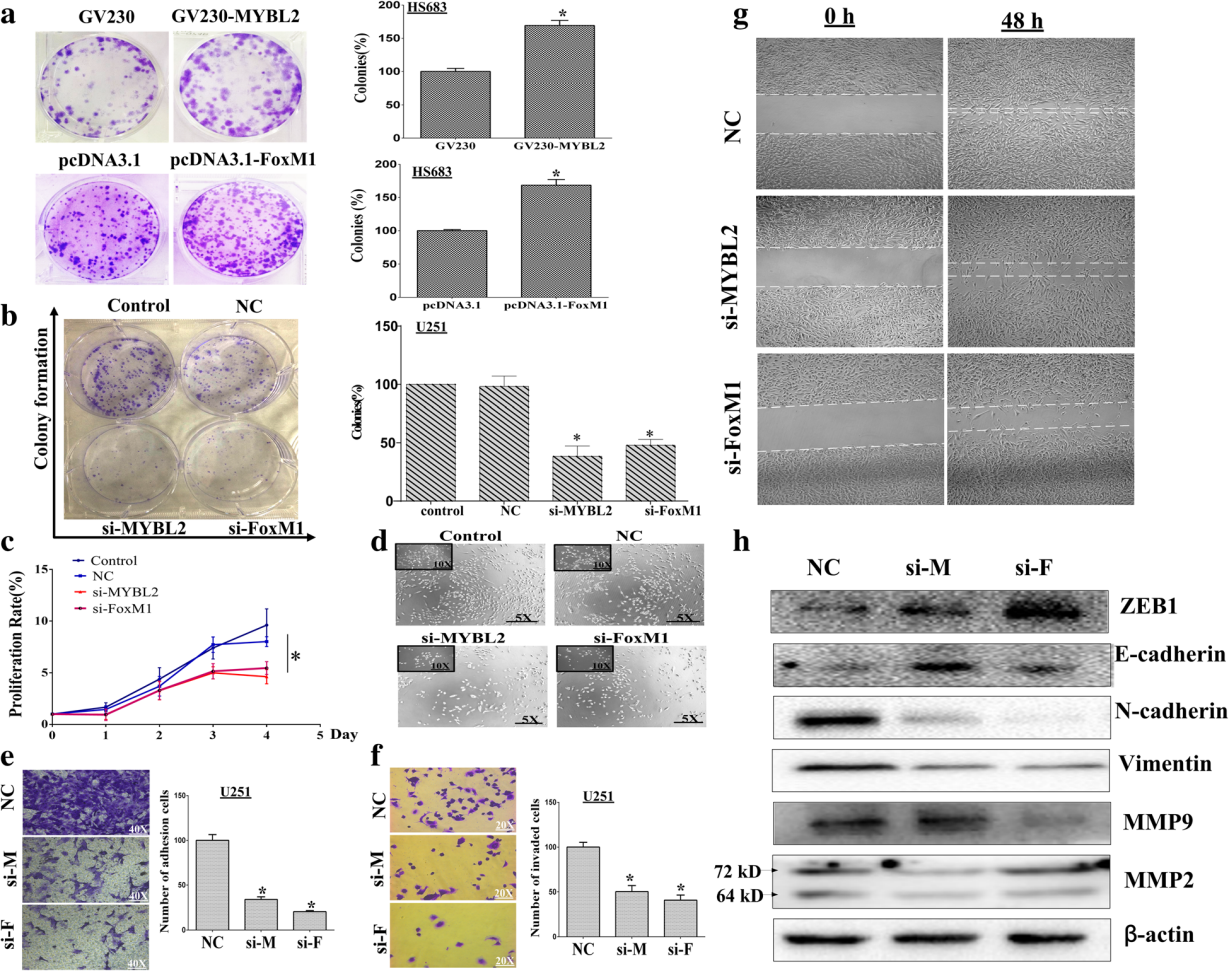
FoxM1 and MYBL2 enhance cancer progression in glioma. **a** Colony formation assays using Hs683 cells, which transfected with GV230-MYBL2 and pcDNA3.1 + HA-FOXM1. **b** Colony formation assays using U251 cells, which transfected with MYBL2-siRNA and FOXM1-siRNA. **c** Effects of MYBL2 and FoxM1 silencing on the proliferation of U251 cells. **d** Cell morphological of U251 cells after silencing MYBL2 and FoxM1. **e** Representative images from transwell migration assays for U251 cells transfected with MYBL2 and FoxM1 siRNA after 48 h. **f** The adhesion of siRNA groups and control group to matrix assessed 2 h after plating. **g** Migration of U251 cells transfected with MYBL2 and FoxM1 siRNAs were identified by wound-healing assays. **h** The effects of MYBL2 and FoxM1 silencing on the expression of EMT markers and MMPs by Western blotting. *p < 0.05

To address the cellular mechanisms of MYBL2 and FoxM1 responsible for tumor progression, MTT assay and colony formation assay were performed. Firstly, we performed foci formation assays as described. In low-grade glioma Hs683 cells, the numbers of colonies were significantly increased by MYBL2 and FoxM1 over-expressing vector (* *p* < 0.05, Fig.4 a.). Conversely, in high-grade glioma U251 cells, the numbers of colonies were reduced by MYBL2 and FoxM1 knockdown (Fig. 4b)**.** Next, we used MTT to assay the proliferation of U251 cells after transfected with siRNAs. U251 cells generally manifest powerful growth ability and was greatly attenuated by knockdown of MYBL2 and FoxM1 in a time dependent manner, especially at 72 and 96 h (*P < 0.05) post transfection of the siRNAs (Fig. 4c). Similar results were found in cell cytomorphology after 48 h down regulation of MYBL2 and FoxM1 (Fig. 4d).

### Decreased MYBL2 and FoxM1 inhibit migration, invasion and EMT of glioma cells

Our results showed that MYBL2 and FoxM1 were both upregulated in human glioma and influenced tumor progression. We further estimate the possible correlations of MYBL2 and FoxM1 expression with metastasis and EMT. In vitro, invasion assay was performed in Boyden chambers with the upper wells coated with Matrigel to mimic the extracellular matrix. As shown in Fig. 4e, the number of cells that passed through a Matrigel coated membrane into the lower chamber was lower in U251cells in silenced groups than NC group (*p < 0.05). Then, the scratch assay was conducted to investigate the effects of MYBL2 and FoxM1 on the migratory behaviors of cells in vitro. The degree of wound healing was assessed every 24 h using a microscope, and representative pictures obtained at 48 h for U251 cells are shown (Fig. 4g). Moreover, we found that cells showed diminished adhesion in both MYBL2 and FoxM1 siRNA groups, and a statistical analysis validated that effects were significant when comparing control cells (Fig. 4f).

Next, we detected the expression of EMT markers (E-cadherin, N-cadherin, ZEB1 and Vimentin) by Western blotting. Both MYBL2 and FoxM1 siRNAs down regulated the protein levels of N-cadherin and Vimentin but increased the levels of E-cadherin and ZEB1 (Fig. 4h).We also detected the effects of MYBL2 and FoxM1 on MMP activity which has been closely correlated with degradation of basement membrane and invasion of cancer cells. The results showed that activity of MMP-2 and MMP-9 were decreased compared with control cells (Fig. 4h).

### Knockout of MYBL2 and FoxM1 reduce expression of G2/M genes and causes delay of cells in G2

**Fig. 5:**
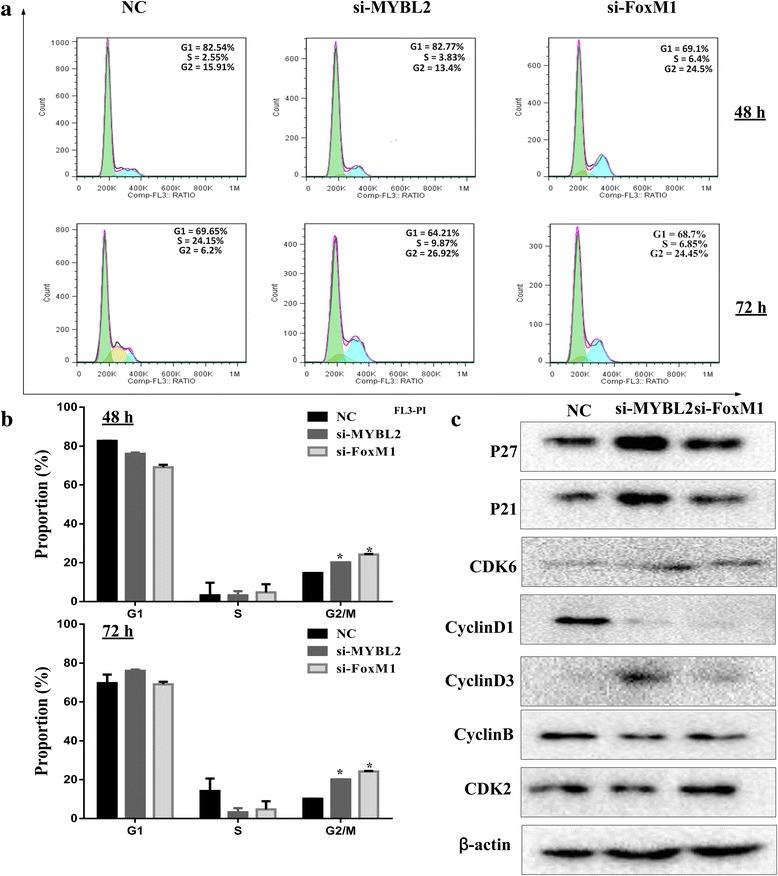
Suppressing MYBL2 and FoxM1 expression inhibited G2/M phase transition in glioma cells. **a** U251 cells were transfected with indicated siRNA, and cells were collected after 48 h. Cell cycle profile was analyzed by flow cytometry. **b** The histogram shows the proportion of cell percentage for 48 h (upper) and 72 h (lower). **c** The Protein levels of cell cycle genes (P21, P27, cyclin D1, cyclin B, CDK2, and CDK6) in U251 cells were detected by Western blot.*P < 0.05

The possible effects of MYBL2 and FoxM1 knockdown on cell cycle progression were assessed by PI staining and flow cytometry. Depletion of MYBL2 and FoxM1 in U251 cells resulted in an increase in cells at the G2/M phase (Fig. 5a and b). The effects of MYB2 and FoxM1 siRNAs on the mRNAs and proteins levels of cell cycle key regulators, including P21, P27, cyclinB1, CDK6, and CDK2 were investigated by Western blotting. As shown in Fig. 5c, the expression of cyclin B and cyclin D down-regulated, but the expression of P21, P27 and CDK6 were up regulated when comparing with NC group. However, MYLB2 and FoxM1silencing have little effect in CDK2 protein (Fig. 5c).

### Down regulation of MYBL2 and FoxM1 induced cell apoptosis in glioma cells

**Fig. 6:**
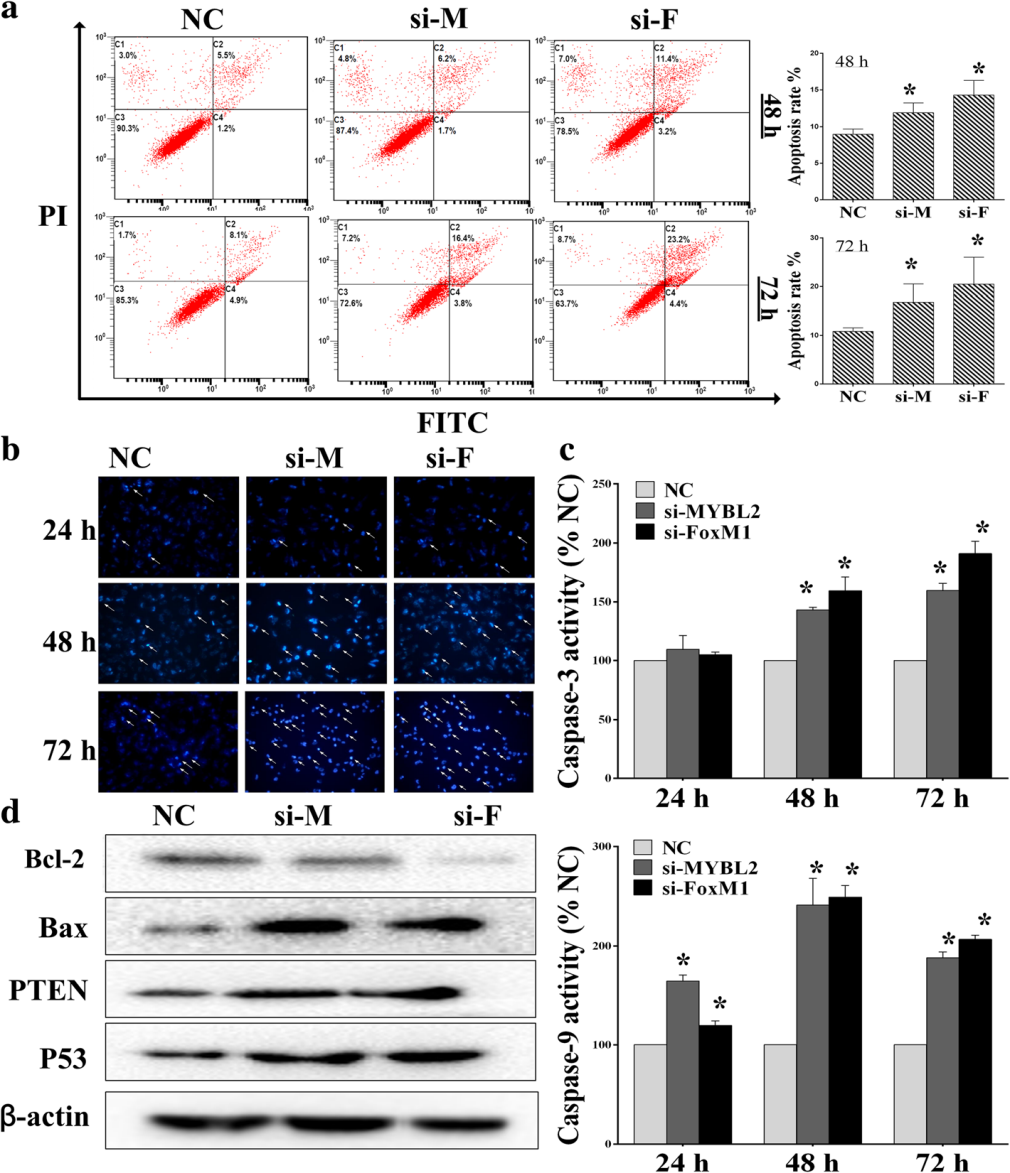
Suppressing MYBL2 and FoxM1 expression induced apoptosis in glioma cells. **a**-**b** Effects of MYBL2 and FoxM1 silencing on the expression of U251 apoptosis by using flow cytometry and Hoechst 3342. **c** Caspase-3/9 activity was tested after MYBL2 and FoxM1 knockdown. **d** The signal protein detecting using Western blotting. *P < 0.05, as compared with NC

To determine whether MYBL2 and FoxM1 are associated with apoptosis, U251 cells were transfected with siRNAs for 24, 48 and 72 h, as described above, the number of apoptotic cells was assessed using an Annexin V-FITC/PI and hochest 3342 staining. As shown in Fig. 6a and b, the percentage of apoptotic cells was increased after 48 h and 72 h. We also tested the effect of MYBL2 and FoxM1 silencing on proteins related to apoptosis including caspase-3/9, Bcl/Bax, PTEN and P53. Western blotting results demonstrated that MYBL2 and FoxM1 down-regulation decreased the expressions of Bcl-2 but increased the expression of Bax. In addition, the protein levels of PTEN and P53 were increased in MYBL2 and FoxM1 siRNAs transfected cells (Fig. 6d). We also conducted caspase-3/9 activity assays and found that knockdown of MYBL2 and FoxM1 induced expression and activity of caspase-3/9 in a time-dependent manner (Fig. 6c).

#### MYBL2 and FoxM1 are co-expression in glioma

**Fig. 7:**
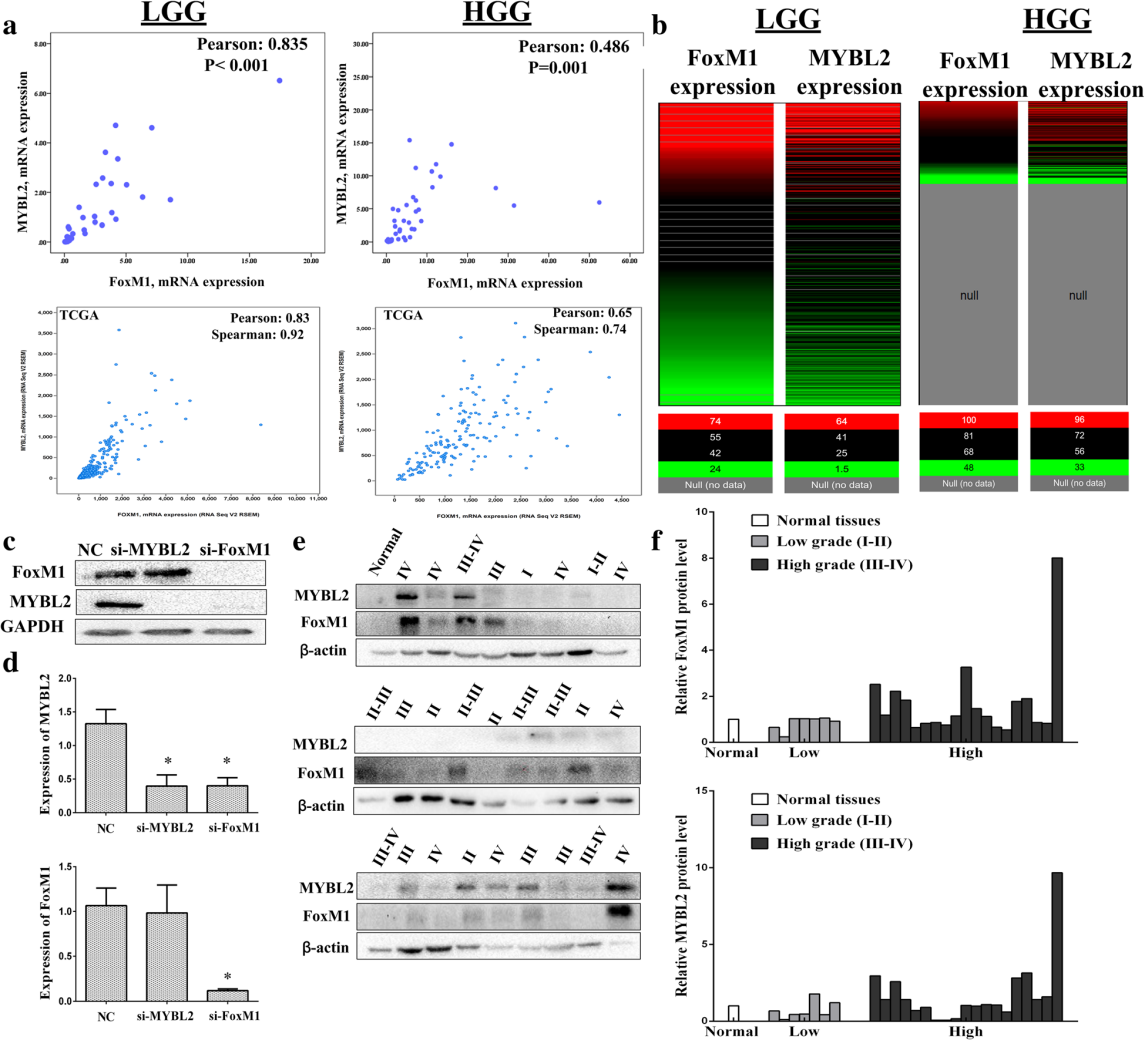
Co-expression of MYBL2 and FoxM1 in glioma. **a** FoxM1 and MYBL2 mRNA expression levels in 79 glioma tissues and TCGA database. **b** The heat map between MYBL2 and FoxM1 in glioma cohort in TCGA database. The analysis was performed by using UCSC Xena. **c**-**d** Western blotting (**c**) and RT-qPCR (**d**) analyze of MYBL2 and FoxM1 expression. **e**-**f** The expression of FoxM1 and MYBL2 were examined by Western blotting in 26 glioma specimens and 1 normal tissue *P < 0.05 represent the protein levels in MYBL2 or FoxM1 group compared to the NC group

Regression analysis showed that MYBL2 and FoxM1 had high correlation coefficients (LGG, *r* = 0.835; HGG, *r* = 0.486; Fig. 7a). Then, we performed separately for high grade and low-grade glioma using cBioPortal. Results showed that whether in low or high-grade glioma, the expression of MYBL2 and FoxM1 are highly correlated (LGG: Pearson’s correlation = 0.83; HGG: Pearson’s correlation = 0.65) (Fig. 7a). In addition, we examined the heap map between MYBL2 and FoxM1 in same data cohort using another tool, the Xena browser (Fig. 7b). To further verify the correlation between MYBL2 and FoxM1, we down regulated both MYBL2 and FoxM1 in U251 cells by siRNAs. As shown in Fig. 7c and d, down regulation of MYBL2 did a little change of FoxM1 expression, while MYBL2 expression was dramatically reduced by knockdown of FoxM1 (*p < 0.05). Moreover, Western blotting analyses showed that MYBL2 and FoxM1 co-expression in protein expression. (Fig. 7 e and f).These results indicated that MYBL2 and FoxM1 had high correlation expression both in mRNA and protein levels.

### Down-regulation of Akt induced FoxM1 and MYBL2 expression

**Fig. 8:**
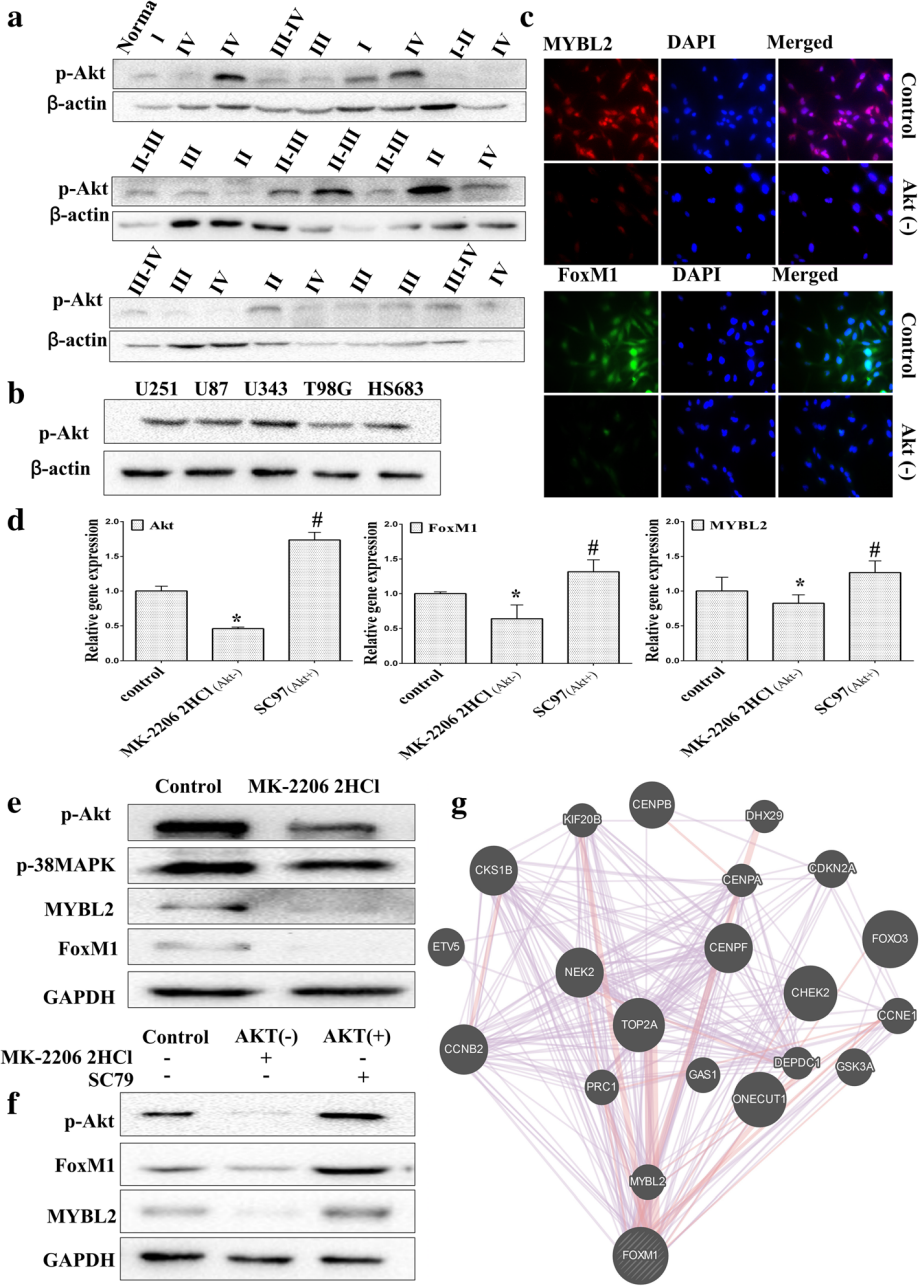
MYBL2 and FoxM1 are activated by Akt signaling pathway. **a** The baseline expression of p-AKT was determined by Western blotting in 26 glioma specimens and 1 normal tissue. **b** The expression of p-Akt was determined in glioma cell lines using Western blotting analysis. **c**-**e** U251 cells were treated with PAMK-2206-2HCL for 24 h. The expression of FoxM1 and MYBL2 were detected by immunofluorescence (**c**) real-time PCR (**d**) and Western blotting (**e**). **f** U251 cells were treated with PAMK-2206-2HCl or SC79 for 24 h. The expression of FoxM1 and MYBL2 were detected by western blotting. **g** The molecular functional network map of canonical pathways including coexpression, physical interaction, and predicted networks of FoxM1 analyzed by GeneMANIA (http://genemania.org/) tool.*P < 0.05 represent MYBL2 group vs. NC group; #*P* < 0.05 represent FoxM1 group vs.NC group

Previous studies showed that FoxM1 was a key downstream gene of the Akt/FoxM1 signaling cascade. Since our results above indicated the correlation of FoxM1 and MYBL2 in glioma progression, we further study the mechanism of MYBL2 and FoxM1 expression in glioma. Therefore, we first tested the basal level of p-Akt in glioma tissues and cell lines using Western blotting. As shown in Fig. 8a and b, over expression of p-Akt was observed in glioma tissues and glioma cell lines. Then, down-regulation of p-Akt by MK-2206 2HCl (Akt inhibitor, 10 nM), we found that Akt inhibitor dramatically inhibited FoxM1 and MYBL2 mRNA and protein expressions in U251cells (Fig. 8c-e). Moreover, we found that SC79 (5 μg/mL), the specific Akt activator, increased the expression of FoxM1 and MYBL2 (Fig. 8f). To further characterize the molecular pathway of FoxM1, the online GeneMANIA tool (http:// www.genemania.org/) was used. MYBL2 and GSK3A (a key regulator of Akt pathway) were confirmed interactions with FoxM1. Take together; these results indicate MYBL2 is a key downstream regulator of Akt/FoxM1 pathway.

**Fig. 9:**
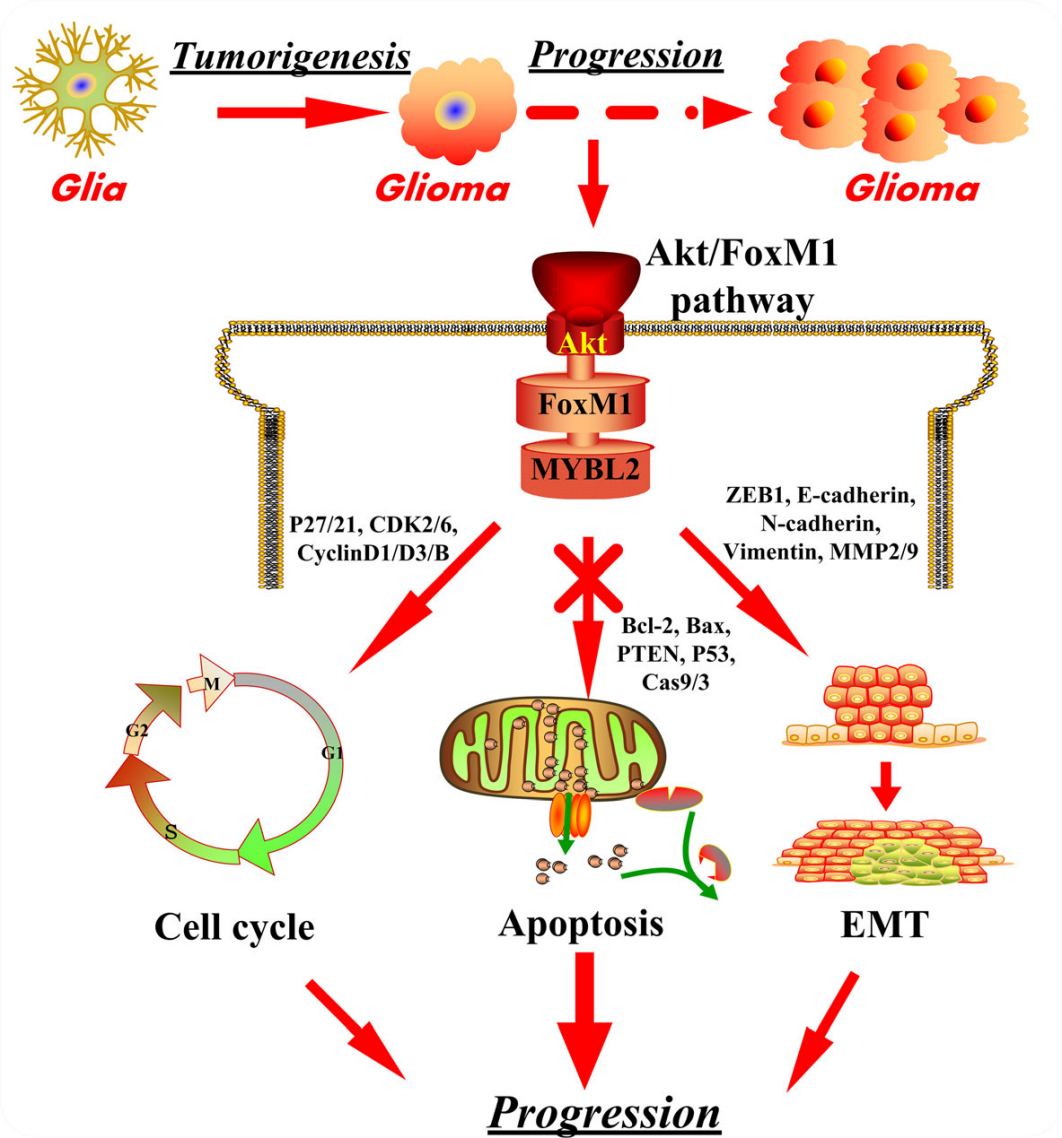
The cartoon depicts the role of MYBL2 and FoxM1 in glioma progression. MYBL2 and FoxM1 act downstream of Akt signaling pathway to regulate the cell proliferation and invasion. And the schematic diagram to illustrate Akt/FoxM1 as the proposed molecular mechanism by which activated FoxM1 up-regulates MYBL2 expression

Our analysis strongly supports that the role of MYBL2 and FoxM1 in glioma progression and illustrates that increased FoxM1 activity up-regulates MYBL2 through the Akt/FoxM1 signaling cascade (Fig. 9).

## Discussion

In the present study, for the first time, we found that MYBL2 and FoxM1 are significantly associated with glioma progress; meanwhile, MYBL2 is interacted with radiotherapy for glioma survival. We also demonstrated the existence of a significant association between MYBL2 and FoxM1 expression in glioma. Furthermore, our results suggested that MYBL2 was downstream of the Akt/ FoxM1 signaling pathway.

Recently, studies found that transcription factors (TFs) and the transcriptional network play important roles in brain tumors progress [25]. In the present study, two transcription factors MYBL2 and FoxM1 emerge as synergistic initiators and master regulators of glioma progress and transformation. We identified both FoxM1 and MYBL2 in high-grade glioma tissues were much higher than in normal brain tissues and low-grade glioma tissues. Either or both over expression of MYBL2 and FoxM1 were associated with poor prognostic.

The postoperative radiotherapy is an established standard treatment for glioma patients [26]. However, glioblastoma is highly resistant to radiotherapy, which is a common problem with current anticancer therapies [27]. So having an individualized radiotherapy plan based on each patient’s radio sensibility is necessary for increasing the treatment efficacy. Thus, the radio sensibility biomarker(s) can be very useful in glioma radiotherapy. The role of FoxM1 in radiotherapy has been reported in GBM [19, 20, 28], but relatively little is known for MYBL2. In this study, we showed that MYBL2 is interacted with radiotherapy for glioma survival. GBM patients, those with MYBL2 high levels without radiotherapy had a significantly higher death risk than those with radiotherapy. Together, these findings further corroborate the rationale of MYBL2 and FoxM1 targeting in combination with irradiation.

Cell cycle progression and epithelial-mesenchymal transition (EMT) are key steps for tumor progress. Previous research had shown that MYBL2 and FoxM1 were both important cell cycle proliferation factors and might collaborate to induce mitosis [29, 30]. To identify the molecular mechanism for the effects of MYBL2 and FoxM1 in glioma progress, we investigated the role of MYBL2 and FoxM1 in cell cycle progression and EMT. The results showed that knockout of MYBL2 and FoxM1 induced a G2/M phase arrest by down-regulation of cyclin B and cyclin D, but up-regulation of P21, P27 and CDK6. In addition, silencing of MYBL2 and FoxM1 down regulated the protein levels of N-cadherin and Vimentin but increased the levels of E-cadherin and ZEB1. These data indicated that MYBL2 and FoxM1 regulators of glioma progress and transformation by inducing cell cycle proliferation and EMT.

The BMYB-FoxM1 complex frequently observed and played an impotent role in cancers with poor prognosis and thought to promote cancer progression by up regulating the expression of mitotic genes [31, 32]. Further study found that MYBL2 is required as a pioneer factor to enable FoxM1 binding to G2/M gene promoters [29]. But, another report showed that a direct transcriptional regulation of FoxM1 by MYBL2, and a feedback loop between the latter and c-Myc, may be governing the replication machinery in ESCs [29, 30]. Consistent with these results, we found that a strong correlation of the co-expression of FoxM1 and MYBL2 were observed in patients with gliomas. To further illuminate the relationship of MYBL2 and FoxM1 in glioma. We knocked down of MYBL2 and FoxM1 by siRNA in glioma cells and found that down-regulation of FoxM1 significant reduced MYBL2 protein expression; while down regulation of MYBL2 did a little change of FoxM1 expression. These results at least partially suggested that MYBL2 was a target of FoxM1 in glioma cells. But additional metadata is required to identify whether MYBL2 expression crucially regulated by FoxM1 through direct interaction with the MYBL2 promoter.

Studies have shown Akt pathway regulate various cell functions, such as angiogenesis, migration and invasion in glioma [33, 34]. Moreover, it is showed that FoxM1 is a key downstream gene in the Akt signaling cascade [21, 33]. In gastric cancer, Akt/FoxM1 signaling has been reported played an important role in chemotherapy [21]. Wang et al. [35] showed that Akt/FoxM1 axis was downstream of CXCL12 and took part in promoting GBM cell invasion. What is interesting is that some researchers reported that there is a positive regulatory feedback loop between FoxM1 and the PDGF/Akt signaling pathway, and the loop promotes breast cancer tumorigenesis [36]. However, another research reported that blocking the Akt pathway by Akt-specific kinase inhibitor did not significantly alter FoxM1B transcriptional activity [37]. Herein, we demonstrated that if Akt can regulate MYBL2 and FoxM1 expression in glioma cells. By knocking down p-Akt expression with Akt inhibitor lowered both FoxM1 and MYBL2 expression, and the activator elevated the two genes expression. Therefore, MYBL2 may be downstream of the Akt/ FoxM1 signaling pathway. Moreover, more studies are needed to see if the feedback loop of FoxM1 and Akt signaling pathway plays a role in MYBL2 expression in glioma.

## Conclusion

In summary, our results suggest that both MYBL2 and FoxM1 over-expression are associated with poor prognosis and EMT in glioma. In cell culture experiments, we find a crosstalk between MYBL2 and the Akt/FoxM1 signaling pathway. The present study raises the possibility that FoxM1 and MYBL2 may be potential targets for cancer therapy as both play crucial roles in glioma progression.

## Additional file

Additional file 1: Table S1. Association of MYBL2 and FoxM1 expression with patient’s clinicopathological features in high grade glioma. (DOCX 19 kb)
