# Correction to: Akt/FoxM1 signaling pathway-mediated upregulation of MYBL2 promotes progression of human glioma

**DOI:** 10.1186/s13046-021-01992-w

**Published:** 2021-06-02

**Authors:** Xue Zhang, Qiao-Li LV, Yuan-Tao Huang, Li-Hua Zhang, Hong-Hao Zhou

**Affiliations:** 1 Department of Clinical Pharmacology, Xiangya Hospital, Central South University, Changsha, 410008 Hunan People’s Republic of China; 2 Hunan Key Laboratory of Pharmacogenetics, Institute of Clinical Pharmacology, Central South University, Changsha, 410078 Hunan People’s Republic of China; 3 Department of Neurology, The Brain Hospital of Hunan Province, Changsha, 410007 Hunan People’s Republic of China

**Correction to: J Exp Clin Cancer Res 36, 105 (2017)**

**https://doi.org/10.1186/s13046-017-0573-6**

Following publication of the original article [1], the authors identified some minor errors in image-typesetting in Fig. 6; specifically in Fig. 6b which displays pictures of Hoechst 3342, a duplicate picture of the si-M was mistakenly used for the si-F.

**Fig. 6:**
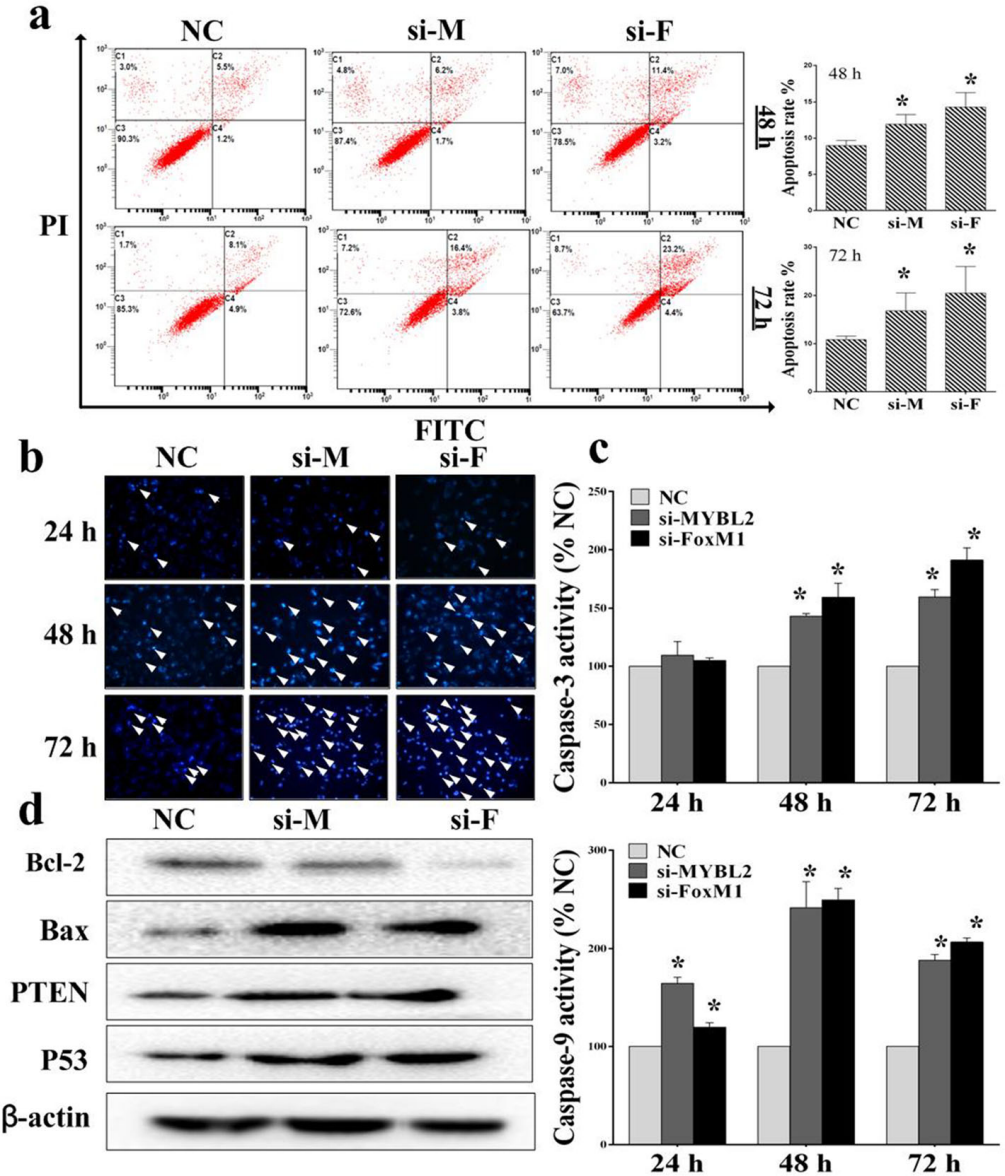
Suppressing MYBL2 and FoxM1 expression induced apoptosis in glioma cells. **a-b** Effects of MYBL2 and FoxM1 silencing on the expression of U251 apoptosis by using flow cytometry and Hoechst 3342. **c** Caspase-3/9 activity was tested after MYBL2 and FoxM1 knockdown. **d** The signal protein detecting using Western blotting. *P < 0.05, as compared with NC

The corrected figure is given below. The correction does not have any effect on the results or conclusions of the paper. The original article has been updated.
