# Supplementary material for: Akt/FoxM1 signaling pathway-mediated upregulation of MYBL2 promotes progression of human glioma
Source: J Exp Clin Cancer Res. 2017 Aug 7;36:105. doi: 10.1186/s13046-017-0573-6 (PMC5547476; doi:10.1186/s13046-017-0573-6)
Supplement: Additional file 1: Table S1. — Association of MYBL2 and FoxM1 expression with patient’s clinicopathological features in high grade glioma. (DOCX 19 kb) [file 13046_2017_573_MOESM1_ESM.docx]

Supplementary Table S1

Table S1. Association of MYBL2 and FoxM1 expression with patient’s clinicopathological features in high grade glioma.

| Feather |  | MYBL2 | | |  | FoxM1 | | |
| --- | --- | --- | --- | --- | --- | --- | --- | --- |
|  | Patients | Low | High | p value |  | Low | High | p value |
| Age |  |  |  |  |  |  |  |  |
| <60 | 270 | 11 | 259 | 0.722 |  | 5 | 265 | 0.482 |
| ≥60 | 297 | 15 | 282 |  |  | 7 | 290 |  |
| Gender |  |  |  |  |  |  |  |  |
| Male | 351 | 20 | 231 | 0.13 |  | 9 | 342 | 0.333 |
| Female | 216 | 6 | 210 |  |  | 3 | 231 |  |
| Rice |  |  |  |  |  |  |  |  |
| White | 498 | 22 | 476 | 0.616 |  | 11 | 487 | 0.648 |
| Others | 69 | 4 | 65 |  |  | 1 | 68 |  |
| Histology |  |  |  |  |  |  |  |  |
| Treated | 32 | 2 | 30 | 0.638 |  | 2 | 30 | 0.127 |
| Untreated | 535 | 24 | 511 |  |  | 10 | 525 |  |

HGG cohorts of TCGA
